# Enhanced optical response of chalcone doped methyl cellulose polymer films for optoelectronic applications

**DOI:** 10.1038/s41598-026-55071-1

**Published:** 2026-05-27

**Authors:** Latha Jogi, Venkatachalam H, Ismayil, P C Dhanush

**Affiliations:** Manipal Institute of Technology, Manipal Academy of Higher Education, Manipal, 576104 Karnataka India

**Keywords:** Chemistry, Materials science, Optics and photonics, Physics

## Abstract

Chalcone doped Magnesium Nitrate: Methyl Cellulose (MCCA) polymer films were prepared through a solution casting approach to examine their structural, optical, and electrical characteristics. Chalcone (CA) was synthesized via Claisen-Schmidt condensation and introduced into the polymer matrix in varying concentrations (0–2 wt%). Structural confirmation of the synthesized compound was carried out using Fourier Transform Infrared Spectroscopy (FTIR), Nuclear Magnetic Resonance (NMR), and mass spectroscopic analyses. FTIR results of the prepared films revealed intermolecular interactions among chalcone, polymer chains, and salt through hydrogen bonding and coordination effects. X-ray diffraction studies indicated the semicrystalline nature of the prepared films. Optical measurements demonstrated a progressive red shift in the absorption edge and a decrease in optical band gap energy with increasing dopant content. Photoluminescence analysis confirmed distinct emission behavior of the modified films. Impedance studies showed ionic conductivity in the order of 10^− 5^ S/cm, indicating that doping mainly enhanced optical properties without significantly affecting ionic transport. These findings demonstrate that chalcone doped MC films possess favourable structural, optical and electrical characteristics suitable for optoelectronic applications.

**Supplementary Information:**

The online version contains supplementary material available at 10.1038/s41598-026-55071-1.

## Introduction

Solid polymer electrolytes have gained significant research interest due to their promising applications in electrochemical and optoelectronic devices such as batteries, sensors, supercapacitors and photonic systems^1, 2^. Compared to conventional liquid electrolytes, solid polymer electrolytes have various advantages including better mechanical stability, flexibility, less leakage risk and superior thermal stability^3, 4, 5^. Polymers have emerged as important materials in modern technology due to their unique combination of physical, mechanical and chemical properties. The multifunctional properties of polymers have been significantly enhanced through the development of Polymer Matrix Composites (PMCs), in which polymers are combined with various fillers or reinforcing agents to improve their strength, stability and overall performance.

Polymer systems incorporated with dopants such as metal salts^6^, nanoparticles^7, 8, 9^, plasticizers^10^, ionic liquids^11^ and organic additives^12^ have been widely reported. Among these, Organic doped polymer systems have drawn considerable attention due to their ability to promote charge transfer interactions, fluorescence behaviour and nonlinear optical properties. Prior research has shown that adding chalcone derivatives to various polymer matrices greatly influence their optical and optoelectronic characteristics. Chalcone doped polymer systems based on blends of PVA (Polyvinyl alcohol) and PVP (Polyvinylpyrrolidone), poly(methyl methacrylate) (PMMA) have been investigated for photonic and nonlinear optical applications^13, 14^. These investigations showed that doping chalcone molecules into polymer matrices reduces optical band gap energy and improves the absorption coefficient, nonlinear refractive index, fluorescence intensity and third order nonlinear optical susceptibility. Specifically, PVA based systems demonstrated increased fluorescence emission and charge transfer interactions between the dopant and polymer matrix, while PMMA based chalcone films demonstrated exceptional nonlinear optical behavior and optical limiting features^15, 16^. Although several synthetic polymer matrices have been studied for chalcone inclusion, studies employing biodegradable methyl cellulose based chalcone doped polymer electrolytes remain very limited.

Among various biodegradable polymers, methyl cellulose (MC) has emerged as a viable host polymer due to its good mechanical property, optical transparency and film forming capability. It is a naturally occurring organic polymer produced by methylating alkali cellulose that offers a good electrode-electrolyte contact in addition to being non-toxic, renewable, widely accessible, inexpensive and biocompatible^17, 18^. The abundant hydroxyl and methoxy functional groups present in methyl cellulose promote intermolecular interactions and ion coordination, which aid in electrolyte production and charge transport^19^. Magnesium nitrate can be considered an effective ionic salt because Mg^2+^ ions interact with the oxygen containing functional groups of methyl cellulose, thereby changing the structure of the polymer chain and promoting ion transport^20, 21^. However, for advanced optoelectronic applications, the optical response of polymer electrolytes based on methyl cellulose still needs to be improved.

Various additives including pyridine^22^, thioureas^23^, thiophene^24^, nitrogen containing compounds^25^ and carboxylic acid groups^26^ have been widely used in polymer matrix as additives to improve their performance in a wide range of electrochemical and energy storage devices^27^. Among various organic compounds, chalcone is an aromatic ketone consisting of two aromatic rings linked through an α-β unsaturated carbonyl bridge^28^. These yellow to orange polyphenolic chemicals can be synthesized through Claisen-Schmidt condensation reaction^29,30, 31^. Chalcones have appealing optical and photophysical characteristics including fluorescence emission, nonlinear optical response and charge transfer characteristics because of their prolonged electron delocalization^32,33,34^. As organic molecules, chalcones cannot be utilized directly in practical photonics device applications due to the chance of degradation when exposed to intense optical signals. They can be doped into a polymer to overcome these limitations make these materials more useful in devices. Doping improves mechanical and thermal properties^35,32^. In polymer electrolyte systems, chalcone molecules are expected to interact with methyl cellulose via hydrogen bonding between the hydroxyl/carbonyl groups of chalcone and hydroxyl/methoxy groups of the polymer. These intermolecular interactions can influence crystallinity, electronic delocalization, optical absorption and fluorescence behaviour of the films. It is proven that the doping of organic materials into the polymer matrix would be of great interest in improving nonlinear optical properties^34^. Chalcone derivatives embedded in polymer electrolytes exhibit excellent optical properties. Chalcone derivatives embedded in polymer electrolytes exhibit excellent optical properties including polarization, colour change, fluorescent complex formation. Polymorphism. Molecular refraction and photochromism^29^.

Detailed research on chalcone incorporated methyl cellulose-magnesium nitrate polymer electrolyte films has not yet been reported, despite the fact that various studies have described methyl cellulose based polymer electrolytes and chalcone doped polymer systems independently. In the present work, chalcone doped methyl cellulose-magnesium nitrate polymer films were prepared using the solution casting technique. The impact of chalcone incorporation on the structural, optical and electrical properties of the films was systematically investigated using FTIR, XRD, UV-visible spectroscopy, fluorescence spectroscopy and impedance analysis. The findings provide insights into the correlation between intermolecular interactions, structural modifications and functional properties of the prepared films for potential optoelectronic applications.

## Experimental

### Materials used

2-Hydroxyacetophenone (Molecular weight: 136.15 g/mol; Purity: 98%) was purchased from Avra Synthesis Private Limited, Telengana. 4-(Methylthio)benzaldehyde (Molecular weight: 152.21 g/mol; Purity: 97.0%) was purchased from TCI, Japan. Sodium hydroxide pellets (Molecular weight: 40 g/mol; Purity: 98%) was procured from Molychem, Mumbai. Methyl cellulose (Viscosity: 350–550 cps) and Magnesium nitrate hexahydrate (Molecular weight: 256.41 g/mol) were procured from Loba Chemie Private Limited, Mumbai. All chemicals were used as received without further purification.

### Synthesis of chalcone

**Scheme 1:**
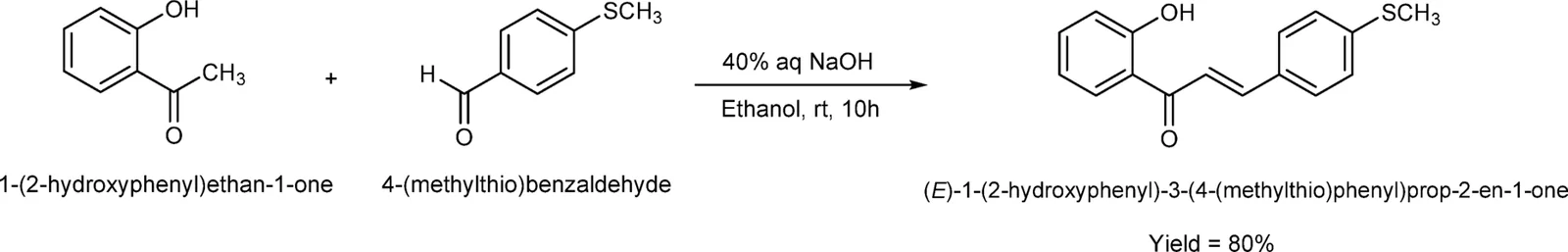
Synthesis of chalcone.

A stirred solution of O-hydroxy acetophenone (0.44 mL, 3.67 mmol) in ethanol was treated with 0.5 mL of 40% aqueous NaOH and allowed to stir for half an hour to form enolate. Subsequently, 4-(methylthio)benzaldehyde (0.38 mL, 3.67mmol) was added dropwise and continued the reaction for 8–10 h at room temperature. Completion of the reaction was confirmed by Thin Layer Chromatography (TLC) using ethyl acetate and hexane (1:9) as mobile phase. The reaction mixture was transferred into a beaker containing ice cold water, neutralized by 4 N HCl. Precipitate formed was allowed to settle and then filtered by vacuum filtration. The solid obtained was dried at 60 °C in hot air oven for 3 h and recrystallized from ethanol^36^. The recrystallized solid was further characterized using FTIR, NMR and mass spectroscopic techniques (Scheme 1).

### Preparation of chalcone doped methyl cellulose based polymer films

Methyl cellulose and magnesium nitrate with weight ratios 75% and 25% were placed in six different beakers and dissolved in N, N-dimethylformamide (DMF). Different compositions of chalcone (0%, 0.25%, 0.5%, 0.75%, 1%, 2%) were then added to the respective solutions. All the mixtures were kept for magnetic stirring at room temperature for about 24 h to obtain a homogeneous mixture. The uniform mixtures were then cast into various petri dishes and kept in hot air oven at 45 °C for the evaporation of the solvent. It took 5–6 days for the complete evaporation of the solvent. The free-standing films were peeled off from a petri dish and characterized using various techniques (Table 1).

**Table 1:** Designation of chalcone doped methyl cellulose based polymer films.

Designation	Methyl cellulose (mg)	Mg(NO_3_)_2_.6H_2_O(mg)	Chalcone (mg)
MCCA0	750	250	-
MCCA0.25	750	250	2.5
MCCA0.5	750	250	5
MCCA0.75	750	250	7.5
MCCA1	750	250	10
MCCA2	750	250	20

### Characterization techniques

The synthesized chalcone was characterized by Shimadzu ATR-FTIR, Bruker NMR spectrometer operating at 400 MHz and mass spectroscopic techniques. The polymer films were characterized by various techniques. A Shimadzu ATR-FTIR spectrometer with a resolution of 4 cm^− 1^ was used to perform FTIR analysis in the wavenumber range of 400 cm^-^^1^ to 4000 cm^− 1^. Film thickness was measured using a Mitutoyo digital screw gauge. XRD diffractograms were obtained using a Rigaku MiniFlex600-C at 2θ angles ranging from 5 to 90° at a rate of 2° per minute. XRD deconvolution was carried out using Fityk software. Absorption spectroscopy was recorded using a Shimadzu UV-1900 I instrument and optical band gap energy was obtained by Tauc’s plot. Fluorescence spectroscopy was recorded using Horiba Fluoromax_Plus_C spectrofluorometer. The CIE chromaticity coordinates were obtained from the fluorescence data using the chromaticity tool in Origin software, employing CIE 1931 color space. Ionic conductivity of the films was measured using a Model ZM 2376 NF LCR meter. I-t characterisation was carried out using Keithley 2450 SMU.

## Results and discussion

### FTIR studies

**Fig. 1:**
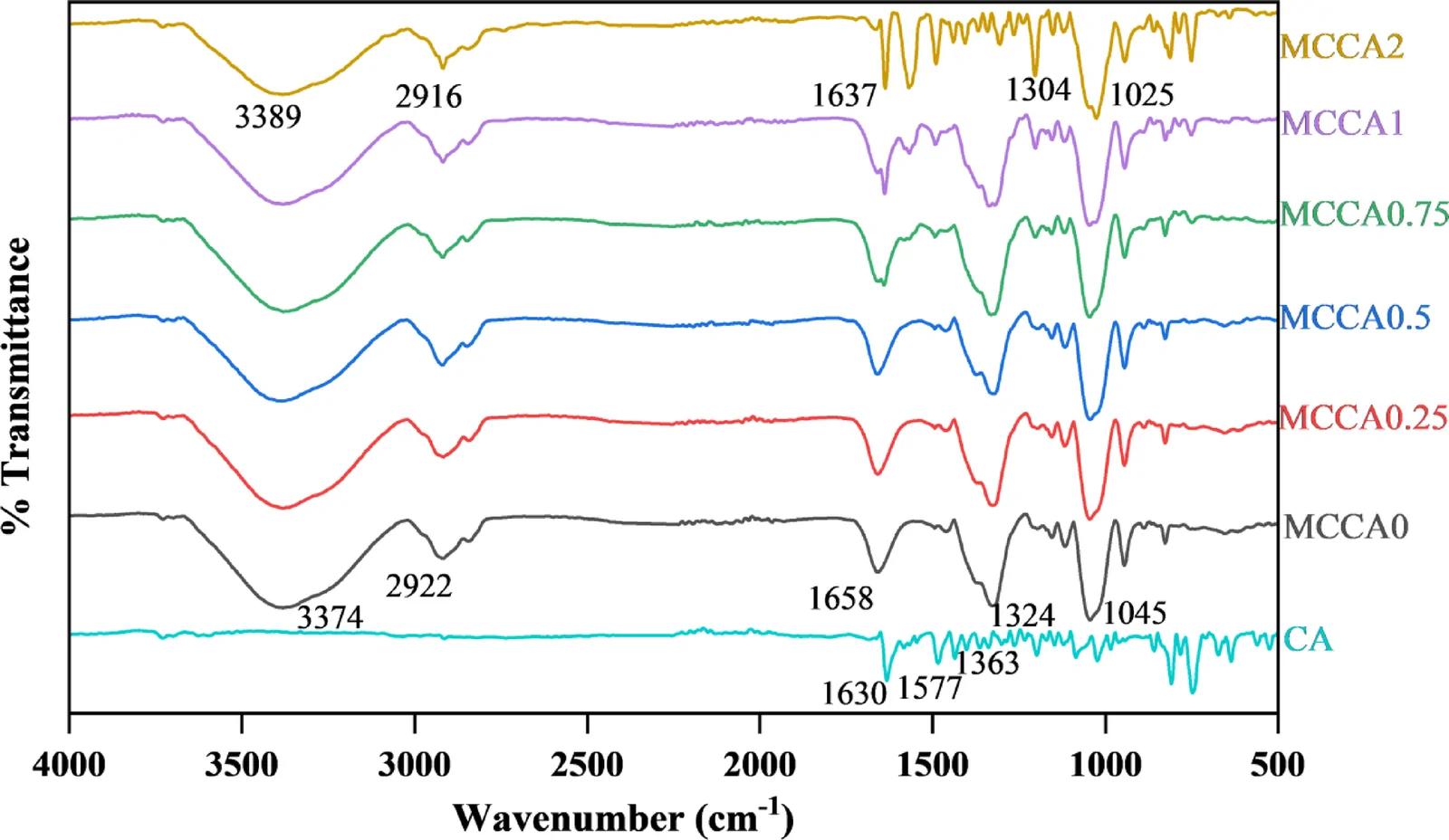
FTIR spectra of pure and chalcone doped polymer films.

The interaction between the chalcone, methyl cellulose and salt was analysed using FTIR spectral studies. The peaks at 3374 cm^− 1^, 2922 cm^− 1^, 1324 cm^− 1^ and 1045 cm^− 1^ correspond to the O-H stretching, C-H stretching, C-H bending and C-O stretching of methyl cellulose respectively^20, 21^. Shifts exceeding 4 cm^− 1^ are considered significant, as the resolution of the IR instrument is 4 cm^− 1^. Upon doping with chalcone, O-H stretching frequency of methyl cellulose shifted towards higher wavenumber (3374 cm^− 1^ to 3389 cm^− 1^). With increasing doping concentration C-H bending and C-O stretching vibrations are downshifted. From the spectra the occurrence of the new peak could be seen around 1560 cm^− 1^ in case of MCCA1 and MCCA2, which is most likely due to the mixed effects of C = C stretching of the aromatic ring and the conjugated double bond of the chalcone. This shifts in the wavenumber and formation of new peak confirms the complexation of chalcone and polymer. Mg^2+^ of salt act as Lewis acid and formed coordination bond with the O-H group of the polymer. On the other hand, oxygen of NO_3_^−^ interacts with methoxy hydrogen of methyl cellulose via weak electrostatic attraction rather than a true hydrogen bond^37^. As electron rich species, hydroxyl substitute of the chalcone is expected to form hydrogen bond with the methoxy hydrogen of the polymer. Similarly, carbonyl group of the chalcone interacts with the hydroxyl group of the polymer via hydrogen bond results in the formation of charge transfer complex. Table 2 lists the characteristics peaks of these major functional groups at the corresponding wavenumber in the MCCA-Mg(NO_3_)_2_ system. All these findings reflected in the IR spectra indicates the significant interaction between the chalcone and polymeric material. The FTIR spectra for MCCA polymer films are shown in Fig. 1. Characterization peaks of the MCCA system are listed in the Table 2.

**Table 2:** The FTIR peak assignment for chalcone doped Methyl cellulose polymer films.

Peak assignment	Wavenumber (cm^− 1^)					
	MCCA0	MCCA0.25	MCCA0.5	MCCA0.75	MCCA1	MCCA2
O-H stretching	3374	3375	3378	3379	3384	3389
C-H stretching	2922	2921	2921	2920	2918	2916
C-H bending	1324	1325	1325	1329	1327	1304
O-H bending (H-O-H bending of absorbed water)	1658	1657	1657	1644	1640	1637
C-O stretching	1045	1045	1045	1045	1045	1025

**Fig. 2:**
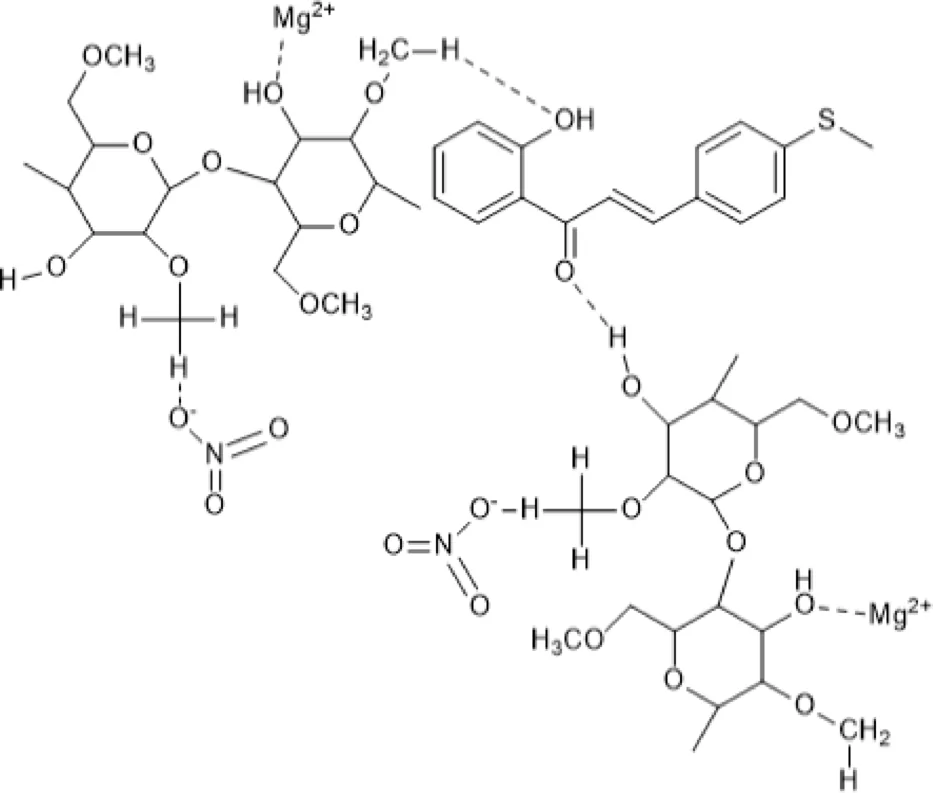
Scheme of probable interaction between the Magnesium Nitrate: Methyl Cellulose and chalcone.

The probable interaction between the chalcone, polymer and salt is shown in Fig. 2.

### X-ray diffraction (XRD)

**Fig. 3:**
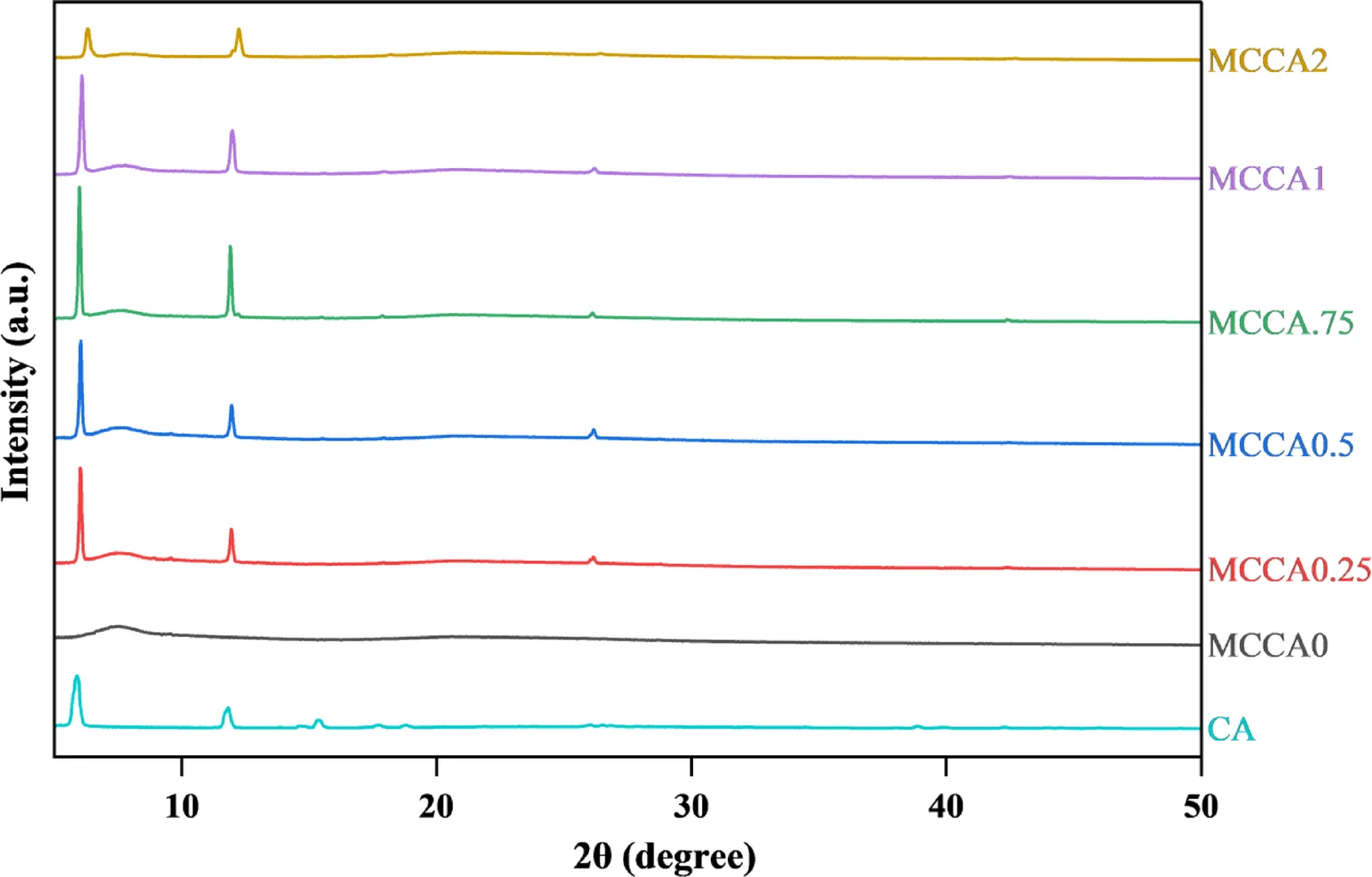
XRD spectra of pure and chalcone doped polymer films.

Structural modifications in the polymer films after doping with chalcone is analysed using X-ray diffraction technique. Pristine methyl cellulose (MCCA0) has a broad diffuse halo with weak intensity in its XRD pattern (around 2θ = 18°-22°)^21^, which is indicative of its amorphous nature resulting from significant hydrogen bonding and disordered polymer chains. After doping with chalcone, appearance of diffraction patterns (centred around 2θ = 8–15°) indicates the introduction of crystalline domains associated with the chalcone molecule. However, the polymer is not transformed into a fully crystalline system rather formed a semi-crystalline structure, where crystalline chalcone domains are scattered within the amorphous polymer backbone. At higher dopant levels (MCCA1 and MCCA2), slight peak broadening and reduced intensity could be observed, which may arise from dopant aggregation and lattice disorder. Overall, XRD demonstrates that chalcone doping improves local ordering in methyl cellulose while retaining its amorphous polymeric nature, which is a favourable characteristic for optoelectronic and flexible functional material applications. Figure 3 represents the XRD pattern of MCCA films.

**Fig. 4:**
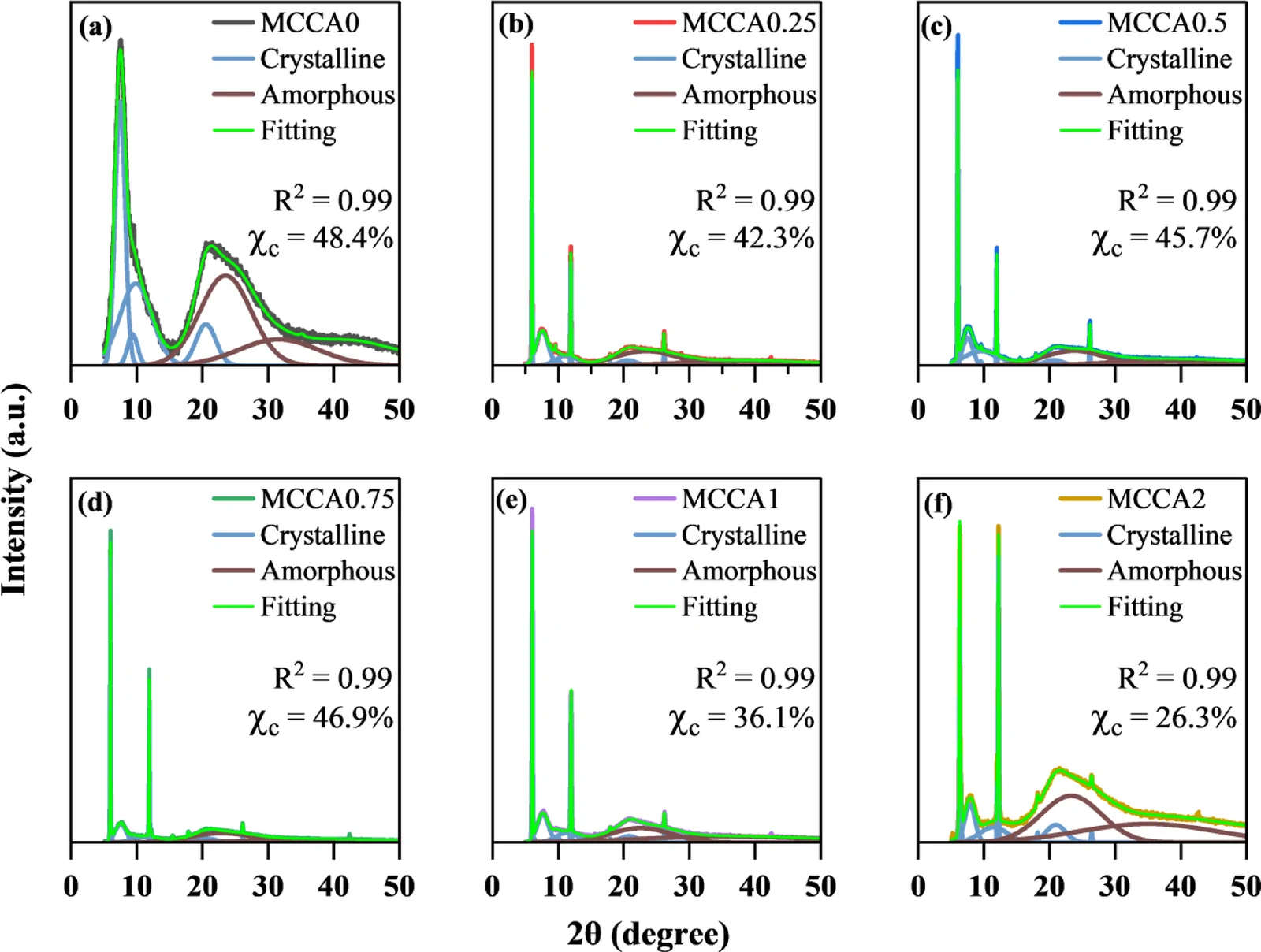
XRD deconvoluted plots of pure and chalcone doped polymer films.

XRD deconvolution was carried out to distinguish the amorphous and crystalline regions of the XRD diffractogram using Fityk software employing Gaussian function. Baseline correction was used prior to the deconvolution process. The following Eq. (1) was used to determine the degree of crystallinity ($\:{\chi\:}_{c}$) of all the films.

1 $$\:{\chi\:}_{c}=\frac{{A}_{c}}{{A}_{A}+{A}_{C}}$$

where A_A_ and A_C_ stand for total area under the amorphous and crystalline peaks respectively. The undoped film exhibits crystallinity of 48.4%, while low chalcone concentrations (0.25–0.75%) preserve comparable results (42–47%), indicating slight rearrangement without considerable disruption of the polymer matrix. The crystallinity significantly drops to 36.1% (MCCA1) and 26.3% (MCCA2) at higher concentrations suggesting that excess chalcone disrupts normal chain packing, suppresses crystal growth and enhances the amorphous proportion. Figure 4 demonstrate the XRD deconvoluted plots of MCCA films.

### UV-visible spectral studies

**Fig. 5:**
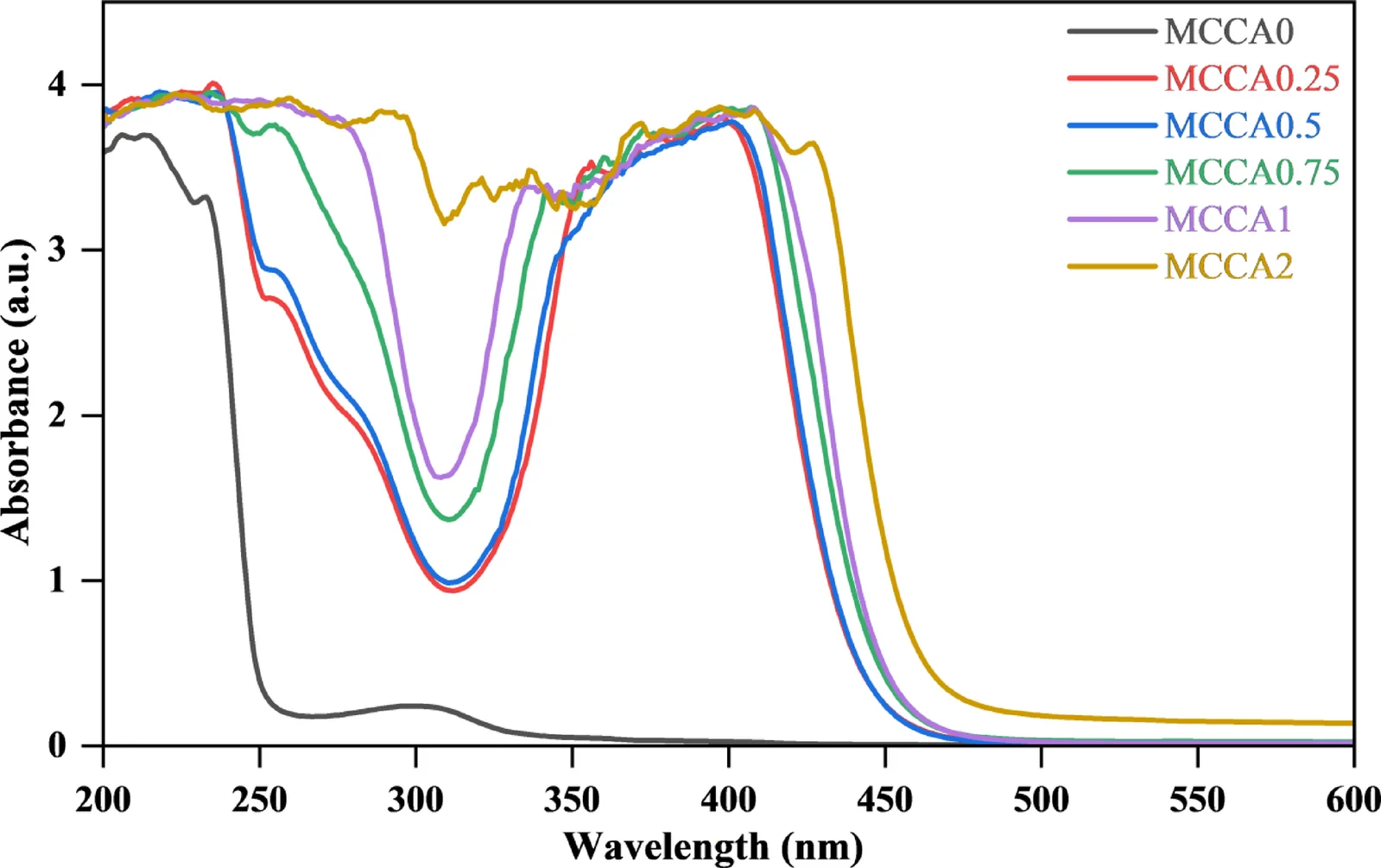
Absorption spectra of pure and chalcone doped polymer films.

Absorption spectra of pure and chalcone doped methyl cellulose films was obtained by UV-vis spectral analysis as shown in the Fig. 5. A shift in the absorption edge was observed for doped polymer when compared to the undoped polymer. With chalcone, the absorbance was increased with the increase of dopant percentage. Maximum absorption was observed around 400 nm, which corresponds to the π-π* transition due to the conjugated π-system of the chalcone^35^. In this region (blue/violet light), the photon energy is close to the materials electronic transition energy, which causes the electrons in the material to strongly interact with the light. As a result, the spectra exhibit a mild red shift in absorption edges, higher absorption and noticeable band broadening indicating enhanced conjugation, significant intermolecular interactions and potential charge-transfer complex formation between the chalcone and methyl cellulose matrix. These modifications point to enhanced electronic delocalization inside the doped films and a decrease in the optical band gap. Such improvements in optical behaviour are advantageous for optoelectronic applications, as higher absorbance and wider light harvesting capacity promote more efficient photon absorption, charge generation and overall photo responsivity. Beyond 500 nm, the light energy is much lower than the energy needed to excite electrons. So, the absorbance decreases slowly and then stabilizes.

### Optical band gap energy and refractive index study

**Fig. 6:**
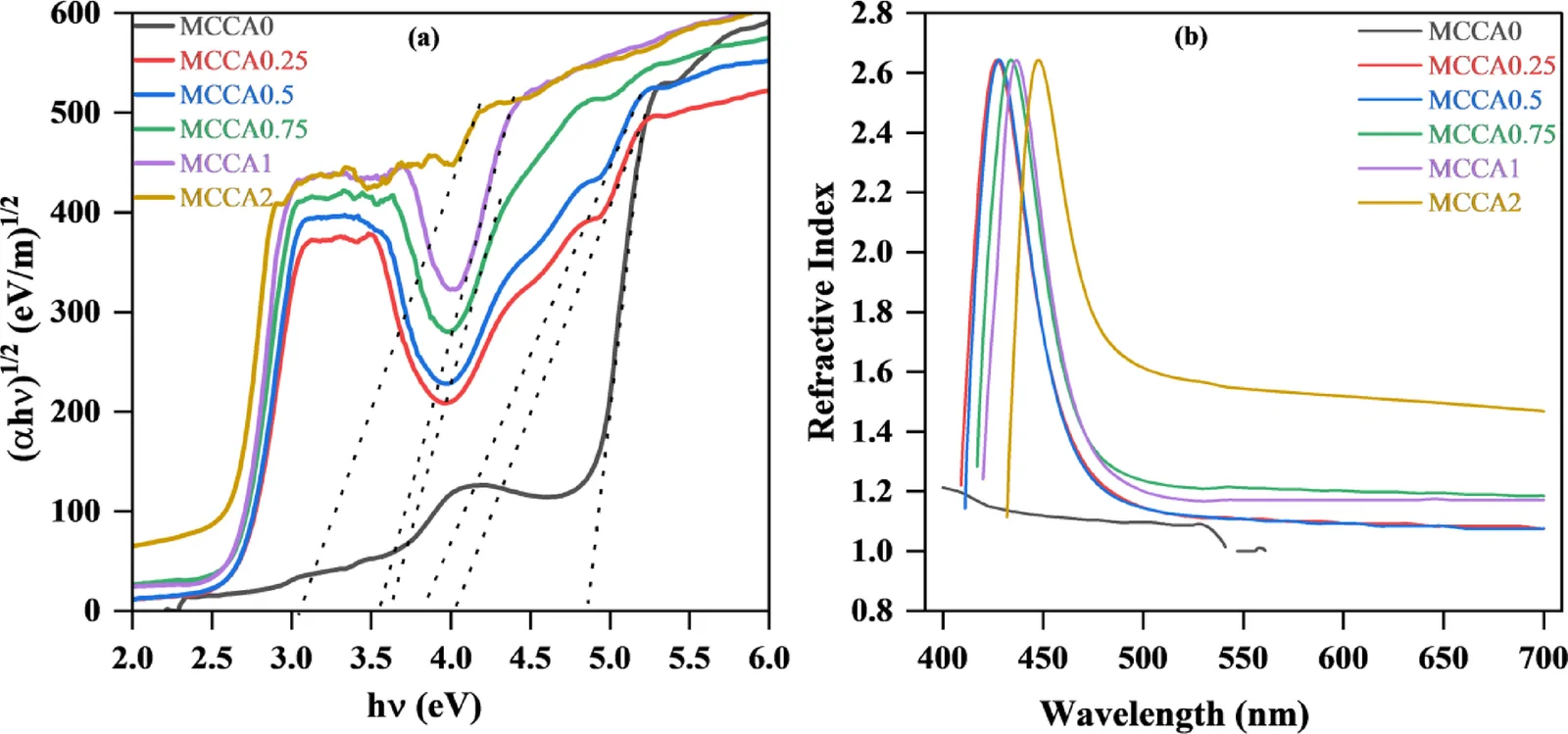
(**a**) Tauc’s plot and (**b**) Refractive Index plot of pure and chalcone doped polymer films.

The minimum energy required for an electron to move from the valence band to the conduction band when a substance absorb light is known as optical band gap energy. This energy band gap (E_g_) of the pure and chalcone doped polymer films was obtained using Tauc’s plot. In Tauc’s plot as shown in Fig. 6. (ahn)^^1/2^ is plotted against the photon energy hυ for a material with an indirect band gap. In doped films, structural disorder and chalcone π-π conjugation introduce localized states within the polymer matrix, which facilitate indirect transition at lower energies. The optical band gap is obtained by extrapolating the linear section of the curve to the energy axis. From the plot, increase in the absorption coefficient and emergence of additional absorption edges in the 3-4.5 eV range could be observed for doped polymer when compared with the pure one. This might be due to the addition of extra energy states within the optical gap during doping. The Tauc extrapolation lines indicate a systematic drop in bandgap with increasing chalcone content, demonstrating enhanced photon absorption in the UV-visible range. The E_g_ values of the films are listed in the Table 2. Change in optical band gap energy with increasing doping concentration is tabulated in the Table 3. The absorption coefficient and optical band gap were estimated using following Eq. 

2 $$\:\alpha\:=\frac{2.303A}{d}$$

3 $$\:{\left(\alpha\:h\upsilon\:\right)\:}^{\frac{1}{n}}\:=B\left(h\upsilon\:-{E}_{g}\right)$$

where ‘a’ is absorption coefficient, ‘h’ is Planks constant and ‘n’ is the frequency of the photon. ‘B’ is a constant, *n* = 2, ‘E_g_’ represents the optical band gap energy^38^.

Figure 6b represents the variation of refractive index with wavelength for MCCA polymer films. Refractive index of all samples increases with chalcone doping compared to pure one. This may be due to enhanced optical density, increased electronic polarization and stronger interaction between the chalcone and polymer matrix. The peak exhibits a slight displacement toward longer wavelengths as the doping concentration increases. This slight red shift may be attributed to the fact that, in blue violet region, the photon energy closely matches the electronic transition energy of the material, leading to strong electron-photon interactions. As a result, the doped films show higher dispersion and sharp increase in refractive index. Beyond 500 nm, light energy is much lower than the energy needed to excite electrons. So, the refractive index decreases slowly and then stabilizes. The observe enhance in refractive index with chalcone doping confirms the reduction in band gap and improved optical response of the polymer films.

**Table 3:** Optical band gap energy of pure and chalcone doped polymer films.

Polymer film	E_g_ (eV)
MCCA0	4.86
MCCA0.25	4.04
MCCA0.5	3.82
MCCA0.75	3.62
MCCA1	3.54
MCCA2	3.06

### Fluorescence spectral studies

**Fig. 7:**
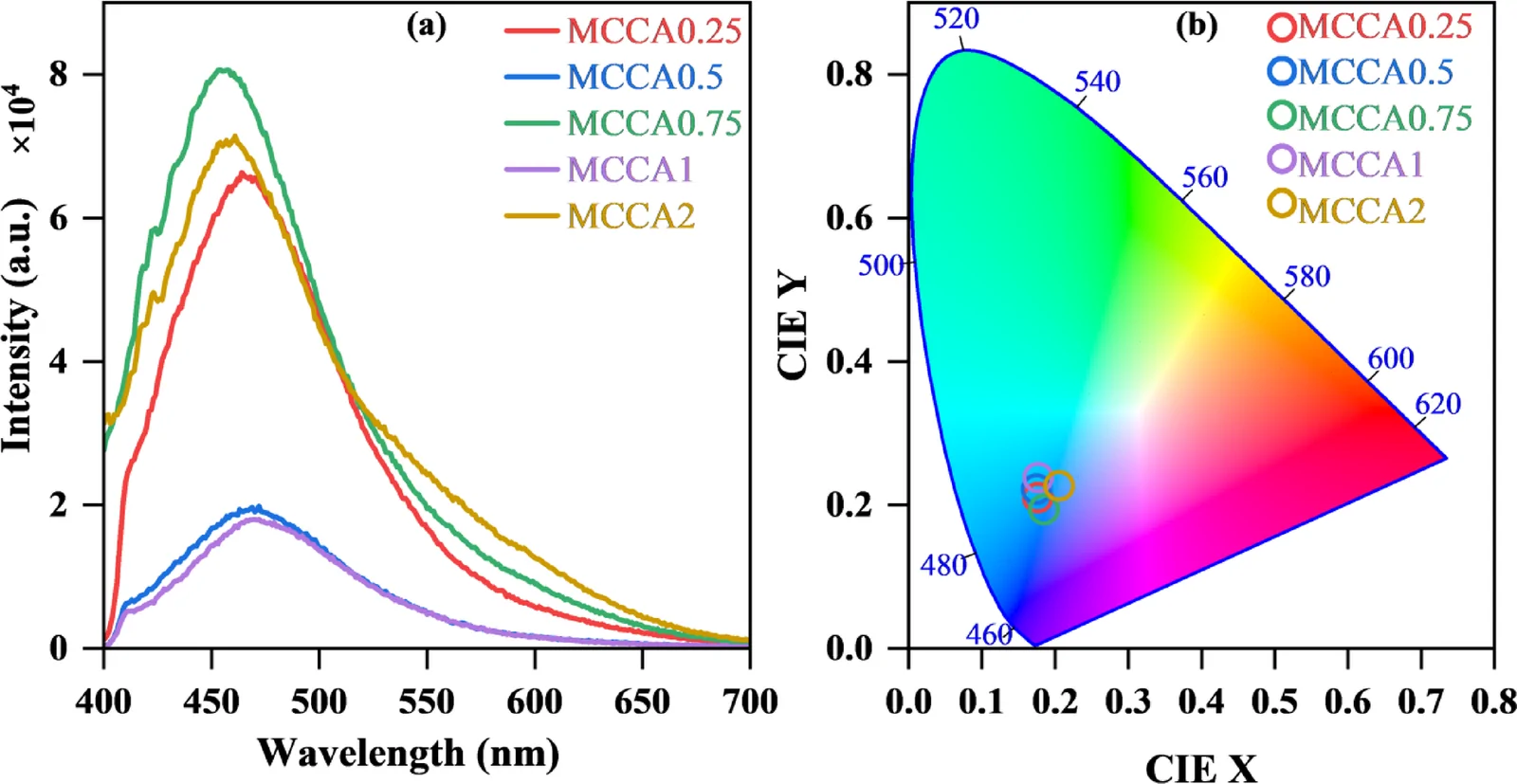
(**a**) Fluorescence spectra of chalcone doped polymer; (**b**) CIE chromatograph of chalcone doped polymer films.

The fluorescence behaviour of the doped polymer was analysed. From Fig. 7a it can be seen that, fluorescence emission wavelength was observed in the range of 480–500 nm for the excitation wavelength of 420 nm. Presence of electron donating groups such as -OH and -SCH_3_ substituents enhances electron density on the aromatic rings thereby promotes intramolecular charge transfer. Additionally, the -OH group stabilizes the excited state via possible intramolecular hydrogen bonding with the carbonyl group which in turn improve molecular planarity and reduce non-radiative relaxation^39^. The α,β-unsaturated chalcone moiety serves as an effective π-conjugated bridge, facilitates charge movement and electron delocalization between the donor and acceptor parts of the molecule. These characteristics produce a donor-π-acceptor system, that promotes intramolecular charge transfer, resulting in strong and consistent blue fluorescence emission.

All the films show a broad emission band in the visible region with no significant shift in peak position. The emission intensity exhibits a non-linear trend, increasing up to MCCA0.75 and then decreasing for higher concentrations (MCCA1 and MCCA2) ,where MCCA0.75 shows the maximum intensity. This enhancement at moderate doping is attributed to the uniform dispersion of chalcone molecules and efficient radiative recombination within the polymer matrix, whereas decrease at higher loading is due to concentration quenching arising from molecular aggregation and enhanced non-radiative decay pathways. These findings imply that methyl cellulose serves as an efficient rigid host for chalcone dyes, with optimal emission achieved at intermediate doping levels.

The CIE chromaticity diagram of the chalcone doped polymer films (Fig. 7b) revealed that all films (MCCA0.25-MCCA2) are situated in the blue-cyan area of the color space, corresponding to emission wavelengths between 460 and 490 nm, which are consistent with the fluorescence spectra. The close clustering of all chromaticity coordinates suggest good color stability with respect to dye concentration. This implies that the chalcone molecule’s excited electronic state is the source of the fluorescence without any additional emissive species. Such stable blue emission with little color change is preferred for optical and photonic applications.

### Impedance analysis

**Fig. 8:**
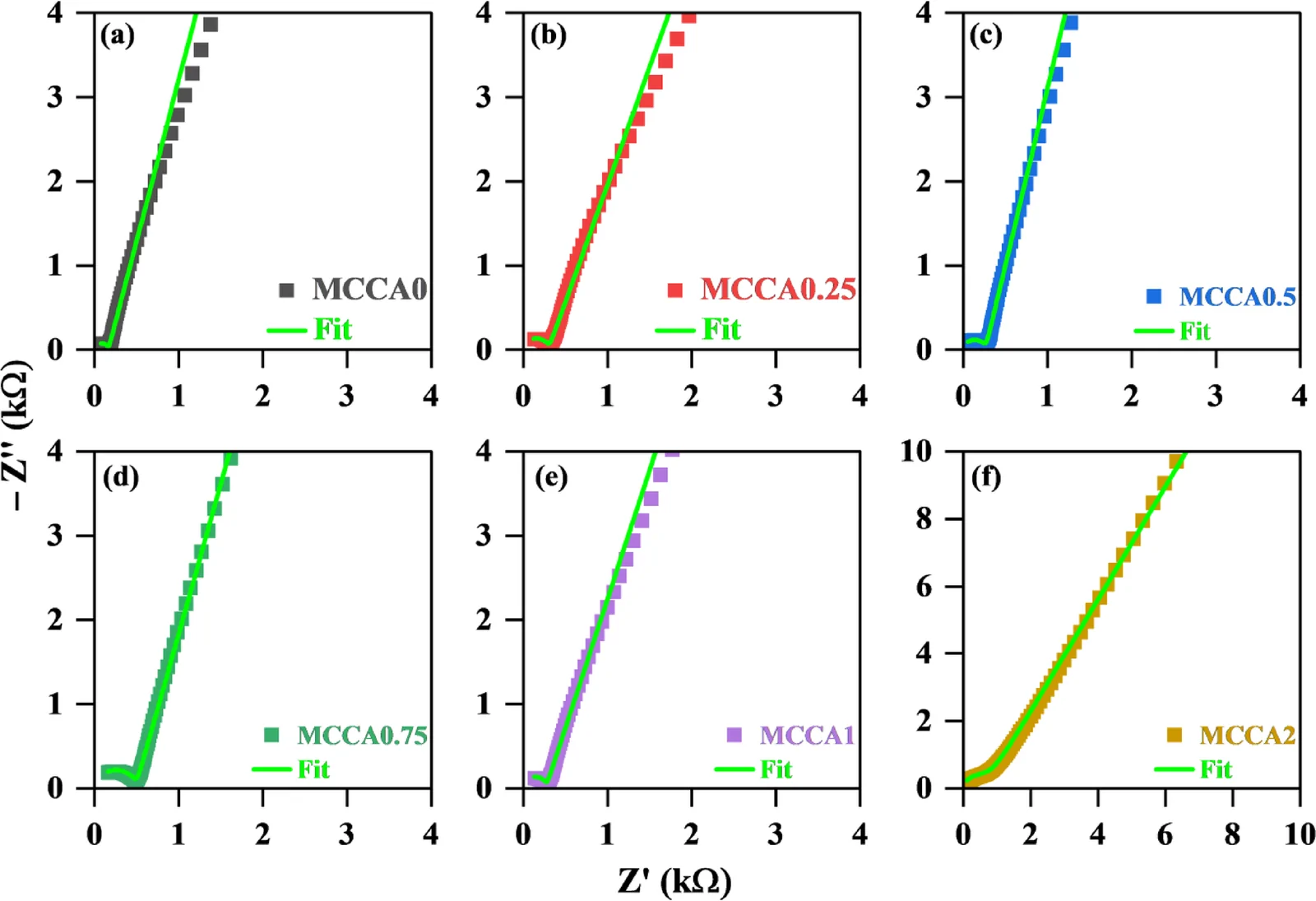
Impedance spectra of pure and chalcone doped polymer films.

The opposition offered by the circuit/ MCCA system to the flow of alternating electrical signal is known as impedance. It is a frequency dependent variable that incorporates resistive and capacitive contributions arising from the polymer matrix, dopant molecules and electrode interfaces. The ionic conductivity of the pure and doped polymer was obtained by impedance spectroscopy. In the Nyquist plot, real part of the impedance (z') reflects the film’s bulk resistance, while imaginary part (z'') corresponds to the energy storage process. Two arcs can be observed in the Fig. 8. The semicircle in the high frequency region represents the bulk resistance and capacitance of the polymer electrolyte. Depressed nature of the semicircle indicates the presence of a constant phase element (CPE) rather than an ideal capacitor. The tilted spike in the low frequency region represents the free charge accumulation at the electrode-electrolyte interphase. The point of intersection of the semicircle with the x-axis indicates the value of bulk resistance^40^. The EIS spectrum analyser was used to fit the Nyquist plots for films. The close overlap between the fitted and experimental curves verifies that the chosen equivalent circuit accurately represents the system’s electrical response. The ionic conductivity of the films was calculated using the following relation.

4 $$\:\sigma\:=\frac{t}{{R}_{b}A}$$

where ‘t’ indicates the thickness of the film, ‘A’ represents the area of the electrode used and ‘R_b_^**’**^ is the electrolyte bulk resistance.

**Table 4:** Values of bulk resistance, thickness and conductivity of pure and chalcone doped polymer films.

Electrolyte	Bulk resistance (Ω)	Thickness (cm)	σ_dc_ (S/cm)	σ_dc_ from AC spectra
MCCA0	165.87	0.01456	1.93 × 10^− 5^	1.60 × 10^− 5^
MCCA0.25	306.07	0.01974	1.42 × 10^− 5^	1.21 × 10^− 5^
MCCA0.5	275.93	0.0174	1.39 × 10^− 5^	1.14 × 10^− 5^
MCCA0.75	492.45	0.01604	0.72 × 10^− 5^	0.61 × 10^− 5^
MCCA1	278.23	0.0144	1.14 × 10^− 5^	0.95 × 10^− 5^
MCCA2	713	0.0146	0.45 × 10^− 5^	0.13 × 10^− 6^

After doping, the order of conductivity remained the same for all the films. All films have shown conductivity in the order of 10^− 5^ S/cm. Doping of chalcone on this polymer electrolyte did not change the ionic conductivity of the film but the optical properties of the films enhanced. Bulk resistance, thickness and conductivity of each film is listed the Table 4.

### Transference number measurement

**Fig. 9:**
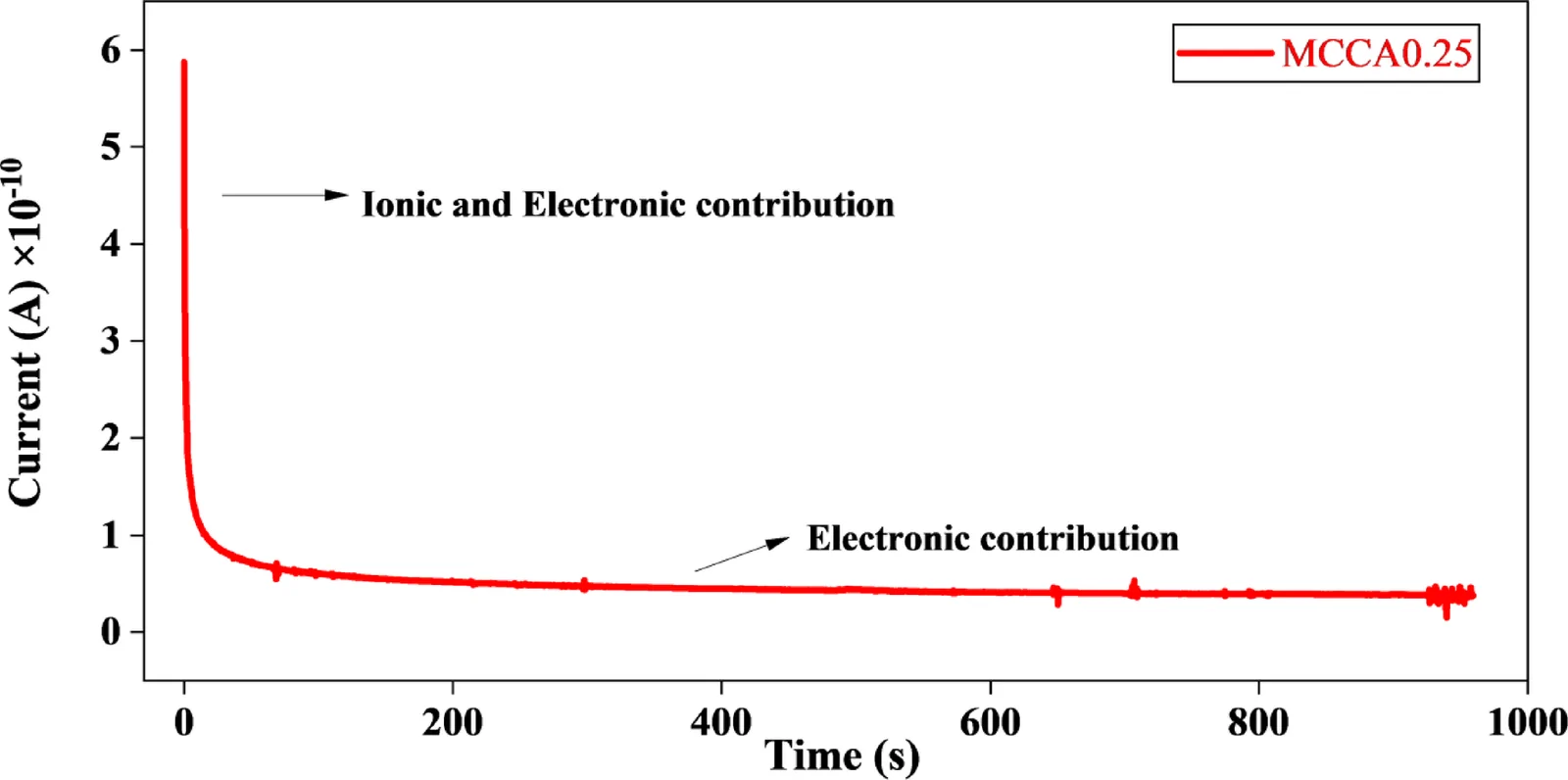
I-t plot of MCCA0.25 polymer film.

The overall conductivity of a solid polymer electrolyte arises from ions and electrons. Transference number measurement helps identify the dominant charge carriers in the electrolyte. I-t spectra of the chalcone doped polymer film (MCCA0.25) was measured at 0.5 V. As shown by Fig. 9. chalcone doped polymer show a sharp initial current at the beginning of the measurement, followed by a rapid decay and then a gradual steady state value. The initial current results from the movement of both ions and electrons. As time passes, the mobile ions build up at the blocking electrode, causing decrease in current. The final steady state current is attributed to electronic contribution. The determined ionic transference number for MCCA0.25 was 0.94 confirms that ions are the majority charge carriers in electrolyte. Following equation is used to calculate the ionic transference number^41^.

5 $$\:{t}_{ion}=1-\frac{{I}_{f}}{{I}_{i}}$$

6 $$\:{t}_{elec}=1-{t}_{ion}$$

Where I_f_ and I_i_ are the initial and final current.

### Study of AC conductivity and dielectric properties

**Fig. 10:**
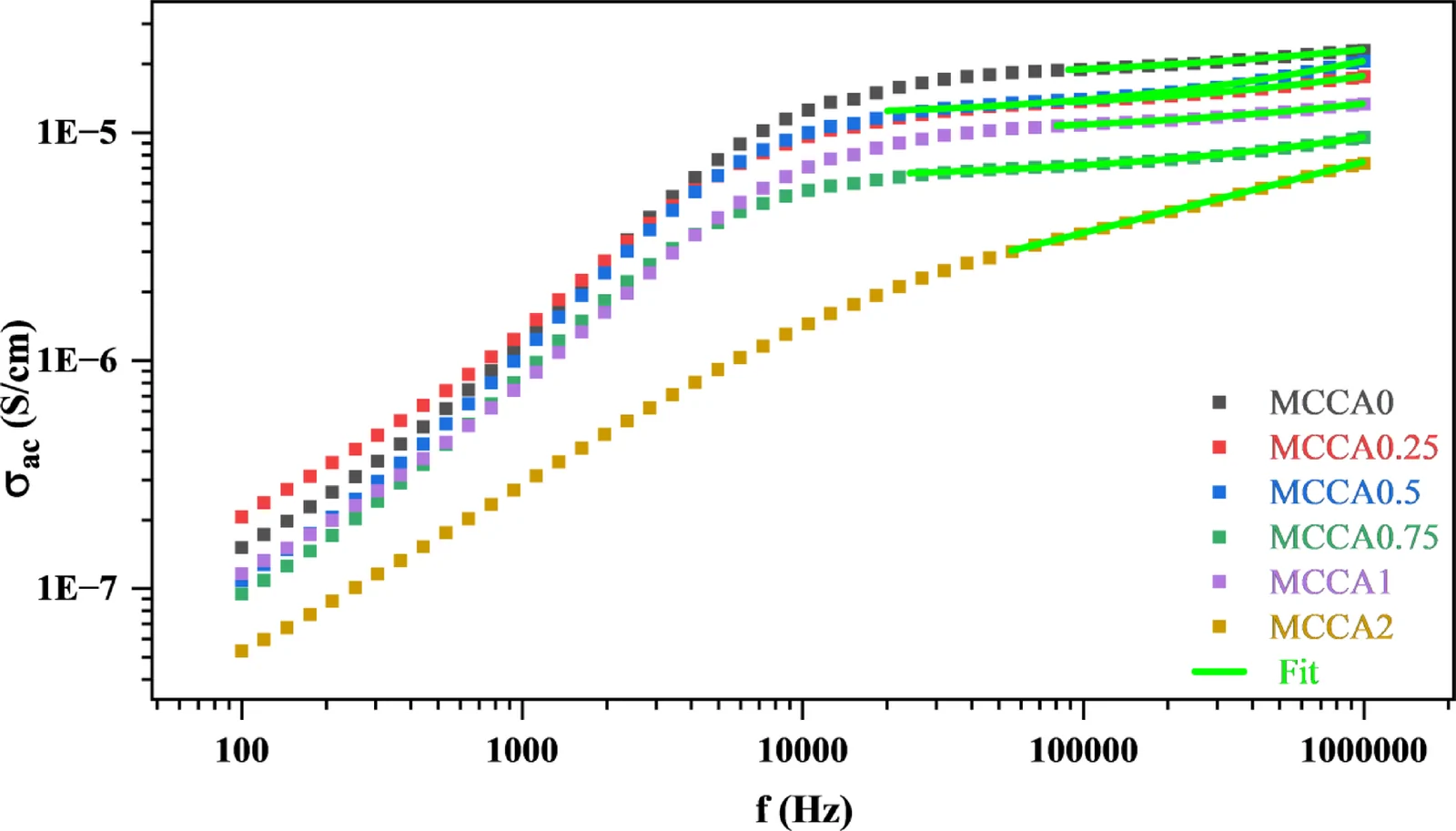
AC conductivity spectra of pure and chalcone doped polymer.

The frequency dependant AC conductivity was computed based on the impedance data using the equation 

7 $$\:{\sigma\:}_{ac\:\:}=\frac{t}{A}\left(\frac{{Z}^{{\prime\:}}}{{Z}^{{\prime\:}2}+{Z}^{{\prime\:}{\prime\:}2}}\right)$$

where ‘t’is the thickness of the film, ‘A’ is the area of the cross section of the electrode used, Z' and Z'' are the real and imaginary part of the complex impedance. As shown by Fig. 10, at low frequencies, the conductivity values are relatively small because mobile ions build up at the electrode-electrolyte interface, producing electrode polarization, which limits their motion. As frequency increases ions can respond more rapidly and contribute to higher conductivity. At high frequencies, the curves tend to level off, forming a plateau that corresponds to the bulk conduction region, where ion motion becomes independent of electrode effects. The fitted line in the plot follows universal power law^42^. This frequency dependent rise suggests that charge transport occurs mainly through hopping conduction, where ions move between localized sites within the polymer matrix. Comparing the different compositions, conductivity decreases with increasing chalcone content. This trend suggests that higher dopant levels introduce structural rigidity and reduce free volume, limiting ionic mobility. Nevertheless, all samples maintain conductivity in the order of 10^− 5^ S/cm. The AC conductivity values calculated are presented in Table 4.

**Fig. 11:**
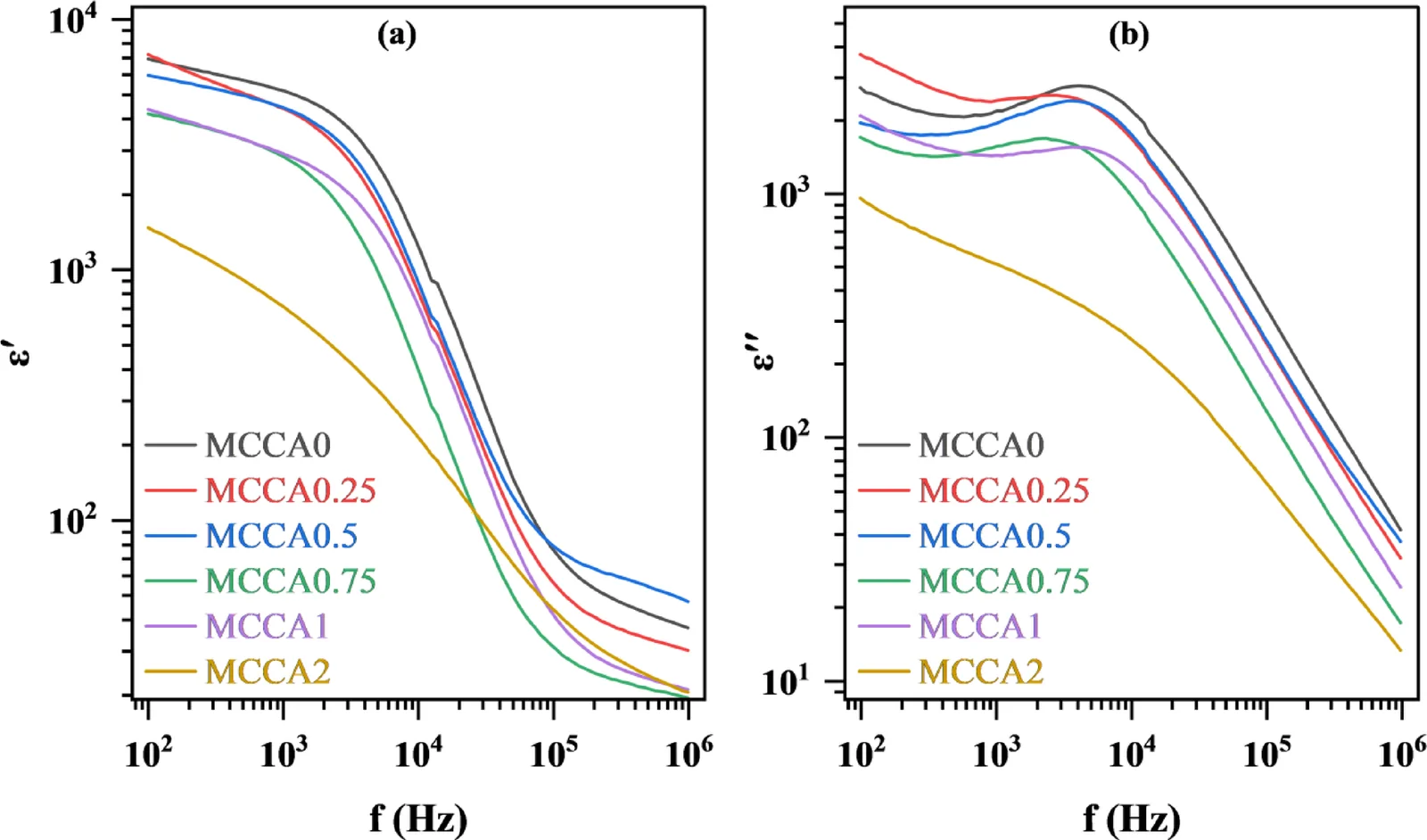
(**a**) Dielectric constant and (**b**) Dielectric loss plot of pure and chalcone doped polymer films.

Figure 11a and b represents the variation of dielectric constant and dielectric loss as a function of frequency respectively. The dielectric constant (ε') and dielectric loss (ε'') of pure and chalcone doped films decrease with increasing frequency. At low frequencies, the high values of ε' and ε'' are due to electrode polarization caused by the accumulation of ions at the electrode-electrolyte interface. With increasing frequency, ions fails to respond rapidly to the alternating field, causing both ε' and ε''to decrease sharply. All the films follow a similar trend. Moderate chalcone concentrations exhibit comparatively higher ϵ'values than the highly doped one. At higher chalcone concentration, agglomeration or restricted polymer chain mobility may reduce the polarization capability, resulting in lower ε' values for MCCA2. ε' and ε'' were calculated based on impedance data from the following equation^43^.

8 $$\varepsilon '~ = ~\frac{{Z^{\prime}}}{{Z^{{'2}} + Z^{{''2}} }}\left( {\frac{t}{{\omega {\rm A}\varepsilon _{0} }}} \right)$$

9 $$\varepsilon ''~ = ~\frac{{Z^{\prime\prime}}}{{Z^{{'2}} + Z^{{''2}} }}\left( {\frac{t}{{\omega {\rm A}\varepsilon _{0} }}} \right)$$

Where is the ω is the angular frequency and ε_0_ is the permittivity of free space.

## Conclusions

Chalcone doped methyl cellulose-magnesium nitrate polymer films were successfully synthesized and characterized by various techniques. FTIR verified the strong interaction between the chalcone and the methyl cellulose. XRD analysis confirmed a semi crystalline nature, where chalcone incorporation modified the polymer ordering and increased the amorphous fraction at higher concentrations. Chalcone doping reduced the crystallinity (48% to 26.3%) and band gap (4.86 to 3.06 eV), reflecting enhanced amorphous nature and localized states. However, ionic conductivity slightly decreases at higher dopant levels, likely due to dopant aggregation and restricted polymer segment mobility, which hinder ion transport despite increased amorphous region. But all the samples retain the conductivity of the order of 10^− 5^ S/cm as undoped one. Optical studies revealed enhanced absorbance, reduced band gap and improved emission characteristics with doping, indicating better photophysical performance, which may be used in optical limiting and photonic device applications.

## Supplementary Information

Supplementary Material 1

## Data Availability

The data that support the findings of this study are available on request from the corresponding author.
