# Supplementary material for: Enhanced optical response of chalcone doped methyl cellulose polymer films for optoelectronic applications
Source: Sci Rep. 2026 May 27;16:24224. doi: 10.1038/s41598-026-55071-1 (PMC13443088; doi:10.1038/s41598-026-55071-1)
Supplement: Supplementary file 1 [file 41598_2026_55071_MOESM1_ESM.docx]

**Supplementary Data**

**Table of contents**

| **Contents** | **Page** |
| --- | --- |
| **Figure S1:** FTIR spectra of chalcone | 1 |
| **Figure S2:** ^1^H-NMR spectra of chalcone | 2 |
| **Figure S3:** ^13^C NMR spectra of chalcone | 2 |
| **Figure S4:** Mass spectra of chalcone | 3 |

**Figure S1:** FTIR spectra of chalcone

 **Figure S2:** ^1^H-NMR of chalcone

**Figure S3:** ^13^C NMR spectra of chalcone

**Figure S4:** Mass spectra of chalcone

**Spectral Analysis**

**(E)-1-(2-hydroxyphenyl)-3-(4-(methylthio)phenyl)prop-2-en-1-one**: Yellow solid; Yield: 80%; M.P: 120°C; FTIR (νcm^-1^): 1632 (C=O), 1567 (C=C), 2914 (C-H ), 3034 (O-H)( **Figure S1**); ^1^H NMR (400 MHz, DMSO, ppm): 12.633 (s, 1H), 8.287-8.266 (m, 1H), 8.039-8.000 (d, 15.6Hz, 1H), 7.882-7.849 (m, 3H), 7.594-7.552 (m, 1H), 7.353-7.332 (d, 8.4 Hz, 2H), 7.030-6.993 (m, 2H,), 2.499 (s, 3H,)( **Figure S2**); ^13^C NMR (100 MHz, DMSO, ppm)δ: 194.02, 162.47, 145.05, 143.16, 136.75, 131.28, 131.24, 130.15, 125.97, 121.39, 121.13, 120.82, 119.59, 118.21, 14.58(**Figure S3**); Molecular formula: C_16_H_14_OS; ESI mass (m/z): 271.16 (M+1)^+^( **Figure S4**).
